# PIRO, SOFA and MEDS Scores in Predicting One-Month Mortality of Sepsis Patients; a Diagnostic Accuracy Study

**Published:** 2019-10-20

**Authors:** Ali Vafaei, Kamran Heydari, Seyed-Saeed Hashemi-Nazari, Neda Izadi, Hassan Hassan Zadeh

**Affiliations:** 1 Department of Emergency Medicine, Shahid Beheshti University of Medical Sciences, Tehran, Iran.; 2 Skull Base Research Center, Loghman Hakim Hospital, Shahid Beheshti University of Medical Sciences, Tehran, Iran.; 3 Prevention of Cardiovascular Disease Research Center, School of Public Health and Safety, Shahid Beheshti University of Medical Sciences, Tehran, Iran.; 4 Student Research Committee, Department of Epidemiology, School of Public Health and Safety, Shahid Beheshti University of Medical Sciences, Tehran, Iran.

**Keywords:** Decision support systems, clinical, patient outcome assessment, mortality, sepsis, shock, septic

## Abstract

**Introduction:**

Different scoring systems based on clinical and laboratory findings are designed for prediction of short-term mortality of patients with severe sepsis and septic shock. This study aimed to compare the screening performance characteristics of PIRO, SOFA and MEDS Scores in predicting one-month mortality of sepsis patients.

**Methods:**

This diagnostic accuracy study was performed on septic shock and severe sepsis patients referring to emergency department of Loghmane Hakim Hospital, Tehran, Iran, from 2017 to 2018. The performance of MEDS, SOFA, and PIRO models in predicting 30-day mortality of patients was evaluated using discrimination and calibration indices.

**Results:**

200 patients with the mean age of 71.03±15.59 years were studied (61% male). During the 30 days, 66 patients died (mortality rate=33%). The area under the ROC curve of PIRO, MEDS, and SOFA scores were 0.83 (95% CI=0.78-0.89), 0.94 (95% CI=0.91-0.97) and 0.87 (95% CI=0.81-0.92), respectively. Based on Brier, Brier_Scaled _and Nagelkerke’s R^2^ of the models, the best performance in predicting one-month mortality belonged to MEDS score. C-statistic showed that MEDS score had the highest value in the differentiation between the survived and non-survived cases.

**Conclusion:**

This study showed that MEDS score performs better than PIRO and SOFA scores in predicting one-month mortality of patients with severe sepsis and septic shock.

## Introduction

Sepsis is the second common cause of mortality among patients in intensive care unit (ICU), and it’s one of the top ten causes of death among all hospitalized patients (1). According to the Centers for Disease Control and Prevention (CDC) reports in United-States, at least 1.7 million people develop sepsis each year. Also, approximately 270,000 Americans die due to sepsis every year (2). Based on World Health Organization (WHO) reports in 2018, burden of sepsis in low-and-middle income countries is highest and sepsis, severe sepsis, and septic shock lead to 20%, 40% and 60% of deaths per year, respectively (3, 4). 

Nowadays, different scoring systems based on clinical and laboratory findings are applied for prediction of short term mortality in patients with critical situations (5, 6). These prediction tools can help the clinicians in selecting the best course of action for the treatment of critically ill patients to get better outcomes. Sequential Organ Failure Assessment (SOFA), Mortality in Emergency Department Sepsis (MEDS), and Predisposition, Infection, Response and Organ dysfunction (PIRO) are three well-known tools for assessment of ill patients with sepsis, severe sepsis and septic shock (7, 8). 

SOFA is an objective and simple scoring system that considers the number and severity of failures in six organs including respiratory system, coagulative function, liver, cardiovascular, kidney, and neurology system. This score ranges from 0 to 24 and higher points predict higher mortality probability. 

The range of PIRO scoring is 0 to 13 (9). Usually rate of respiratory system, bandemia, pulse rate, and temperature are being evaluated and finally for the organ dysfunction, alteration in the mental status, according to the Glasgow Coma Scale (GCS), systolic blood pressure, platelet count, prolongation of prothrombin time and etc. are being considered (10). 

Different studies have compared these scores for predicting mortality, but there is not a general consensus regarding the best and most accurate rule in this regard (11-13). Macdonald et al. reported that PIRO model performed better than SOFA score and similar to MEDS score for predicting mortality in ED patients with severe sepsis and septic shock (14). In addition, in Nguyen’s study, PIRO performed equally well when compared with APACHE II and surpassed MEDS in discriminating survivors from non-survivors (11). Therefore, in this study, we are going to compare the performance measures of SOFA, PIRO, and MEDS scoring models in predicting 30-day mortality of septic shock and severe sepsis patients.

## Methods

***Study design and setting***

In this diagnostic accuracy study, septic shock and severe sepsis patients referring to Loghmane Hakim educational Hospital in Tehran province of Iran from 2017 to 2018 were examined. The performance of MEDS, SOFA, and PIRO models for predicting 30-day mortality were evaluated using discrimination and calibration indices. The protocol of study was approved by ethics committee of Shahid Beheshti University of Medical Sciences (Ethics code: IR.SBMU.MSP.REC.1396.119). The researched adhered to the principals of Helsinki recommendations regarding the ethical consideration in medical researches.

***Participants***

All patients aged > 18 years with septic shock and severe sepsis who were admitted to intensive care unit (ICU) during the study period were examined. Patients with heart attack, pulmonary embolism, cancer, human immunodeficiency virus (HIV) infection, trauma and those who had recent major surgery were excluded. The instructions of scores regarding the included patients' characteristics were considered during data collection. 

***Data gathering***

The necessary information from the clinical examination and medical records were extracted. Data collection form included age, sex, admission ward, duration of admission, transfer type (EMS or private car), history of smoking, opium abuse, history of different underlying diseases (kidney, hypertension, ischemic heart disease, ICU hospitalization in previous 3 months, IV antibiotic therapy in previous 30 days, previous trauma), early and final diagnosis, and vital sign findings including tachycardia, tachypnea, temperature, blood pressure, respiratory rate.

 Every patient was followed for at least one month (30 days). In absent cases, the research staff contacted the patient or patient's family at certain intervals and attempted to collect the necessary medical information of the patient's latest condition.

A third year emergency medicine resident was responsible for data gathering, follow up, and calculation of scores for all patients, under the direct supervision of an emergency medicine specialist.

***Definitions***

- Severe sepsis was defined as having two or more criteria from the "Systemic Inflammatory Response Syndrome (SIRS)", at least one criterion from signs of circulatory shock and one criterion from the evidence of infection (15). 

- Septic shock patients were those diagnosed with systolic blood pressure (SBP) lower than 90 mmHg who did not respond to treatment with at least one liter of Crystalloid serum, and still had SBP<90 mmHg or lactate level ≥4 mmol

***Sequential Organ Failure Assessment (SOFA)***

This score is used during the stay in the ICU and is based on six different indices including: respiration (PaO_2_/FiO_2_ (mmHg) or SaO_2_/FIO_2_ (mmHg)), cardiovascular system (status of hypotension), liver function (bilirubin level (mg/dl) [μmol/L]), coagulation status (platelets count), kidney function (creatinine level or urine output) and neurology status (Glasgow coma scale). SOFA score ranges from 0 to 24 points and higher scores predict higher mortality probability in infected patients (16).

***Mortality in Emergency Department Sepsis (MEDS)***

MEDS score comprises of nine variables, including terminal illness (6 points), septic shock, tachypnea or hypoxemia, platelet count<150,000 cells/mm^3^, bands>5%, age>65 yrs. (3 points for each variable, respectively), lower respiratory infection, nursing home resident, and altered mental status (2 points for each variable, respectively). In this study we used all these variables except bandemia for calculating MEDS score because bandemia was not reported for the patient in the hospital. Hence the range of this score is from 0 to 24 points depending on whether variables were present or absent (17). 

***Predisposition, Infection, Response, and Organ dysfunction (PIRO)***

For calculation of PIRO score we used the first table provided by H. Bryant et al, in their article (11). In this scoring system patients got a score between 0 to 13 according to their age, co-existence of comorbidities like chronic liver disease, congestive cardiomyopathy, existence of community acquired urinary tract infection (UTI) or hospital acquired UTI and also the type of culprit pathogen, presence of tachycardia and tachypnea, and the number of organ failures and also hepatic failure (18).

**Table 1:** Comparing the baseline characteristics of studied patients between survived and non-survived groups

**Variable**	**Survived (n = 134)**	**Died (n = 66)**	**P-value** ^*^
**Demographics**			
Gender (male)	87 (71.31)	35 (28.69)	0.10
Age (year)	66.02 (15.75)	81.18 (8.98)	<0.001
Weight (kg)	68.7 (11.48)	65 (10.32)	0.02
BMI (kg/m^2^)	23.85 (3.28)	23.05 (3.4)	0.10
**ICU admission**			
No	98 (73.13)	36 (26.87)	0.009
Yes	36 (54.55)	30 (45.45)	
**Duration of Admission (day)**			
<5	31 (54.39)	26 (45.61)	0.052
5-10	50 (70.42)	21 (29.58)	
>10	53 (73.61)	19 (26.39)	
**History**			
Living in a nursing home	9 (47.37)	10 (52.63)	0.07
Smoking	41 (69.49)	18 (30.51)	0.62
Cardiovascular failure	8 (44.44)	10 (55.56)	0.03
Previous trauma	12 (57.14)	9 (42.86)	0.31
Kidney diseases	20 (68.97)	9 (31.03)	<0.001
End stage disease	68 (57.63)	50 (42.37)	0.001
Hypertension	89 (63.57)	51 (36.43)	0.11
**Ischemic Heart Disease **	26 (50)	26 (50)	0.002
**ICU admission (3 month ago)**	43 (64.18)	24 (35.82)	0.54
**Serum Lactate level**			
<2	64 (92.75)	5 (7.25)	<0.001
2-2.9	45 (86.54)	7 (13.46)	
3-3.9	6 (60.00)	4 (40.00)	
≥4	19 (27.54)	50 (72.46)	
**Drug history**			
Steroids	11 (61.11)	7 (38.89)	0.57
Beta blocker	34 (51.52)	32 (48.48)	0.001
Opium	25 (69.44)	11 (30.56)	0.73
IV Antibiotic (> 30 days ago)	47 (54.65)	39 (45.35)	0.001
**Vital signs / SIRS**			
Tachycardia	107 (63.69)	61 (36.31)	0.02
Tachypnea	93 (64.58)	51 (35.42)	0.24
Temperature (>38 or <35.5)	110 (65.87)	57 (43.13)	0.44
SBP<90 mmHg or MAP<70	17 (27.42)	45 (72.58)	<0.001
WBC (>15000 or <4000)	84 (62.69)	50 (37.31)	0.06
Respiratory rate > 20	95 (62.91)	56 (37.09)	0.03
Acidosis	89 (64.03)	50 (35.97)	0.17
Platelet < 150000	38 (56.72)	29 (43.28)	0.02
Septic Shock	21 (29.58)	50 (70.42)	< 0.001

Data are presented based of mean ± standard deviation or frequency (%).BMI=Body Mass Index; ICU: intensive care unit; IV: intravenous; SIRS: systemic inflammatory response syndrome; SBP: systolic blood pressure; MAP: mean arterial pressure; WBC: white blood cell count.

**Table 2:** Comparing the baseline characteristics of studied patients between survived and non-survived groups (continue)

**Variables**	**Survived (n = 134)**	**Died (n = 66)**	P-value*
**Scores**			
PIRO	5.08 ± 2.18	8.01 ± 1.9	<0.001^*^
MEDS	8 (7 - 10)	16 (13 - 17)	<0.001^**^
SOFA	3 (3 - 5)	7 (6 - 9)	<0.001^**^
**Source of infection**			
Urosepsis	35 (66.04)	18 (33.96)	0.86
Wound sepsis	15 (83.33)	3 (16.67)	0.12
Pneumosepsis	35 (49.3)	36 (50.7)	<0.001
Others	49 (84.48)	9 (15.52)	0.001
**Peripheral blood smear**			
Gram negative	42 (61.76)	26 (38.24)	0.25
Gram positive	15 (57.69)	11 (42.31)	0.27
**Transfer type**			
Own car	57 (69.51)	25 (30.49)	0.52
EMS	77 (65.25)	41 (34.75)	

Data are presented as mean ± standard deviation, median (Q1 - Q3) and frequency (%); PIRO: Predisposition Insult Response and Organ; MEDS: Mortality in Emergency Department Sepsis; SOFA: Sequential Organ Failure Assessment. *Based on t-test; ** Based on Mann-Whitney test.

**Table 3:** Performance characteristics of PIRO, MEDS, and SOFA scores in predicting 30-day mortality of sepsis patients

**Characteristics**	**PIRO**	**PIRO** *	**MEDS**	MEDS*	**SOFA**	SOFA*
**Overall**						
Brier	0.153	0.156	0.086	0.089	0.128	0.132
Brier_Scaled_ (%)	30	30	61	61	41	41
R^2 ^(Nagelkerke)(%)	41	40.9	71.5	70.9	50	49.3
**Discrimination**						
C-Statistic	0.835	0.836	0.941	94	0.872	0.872
Slope	0.31	-	0.62	-	0.42	-
**Calibration**						
In- the-large	0	0.07	0	0.01	0	-0.01
Slope	1	1	1	0.98	1	0.99
H-L tests, χ^2^ (P)	2.92(0.93)	-	4.82(0.77)	-	7.03(0.53)	-

H-L=Hosmer-Lemeshow; *Optimism Corrected with bootstrap method.

**Figure 1:**
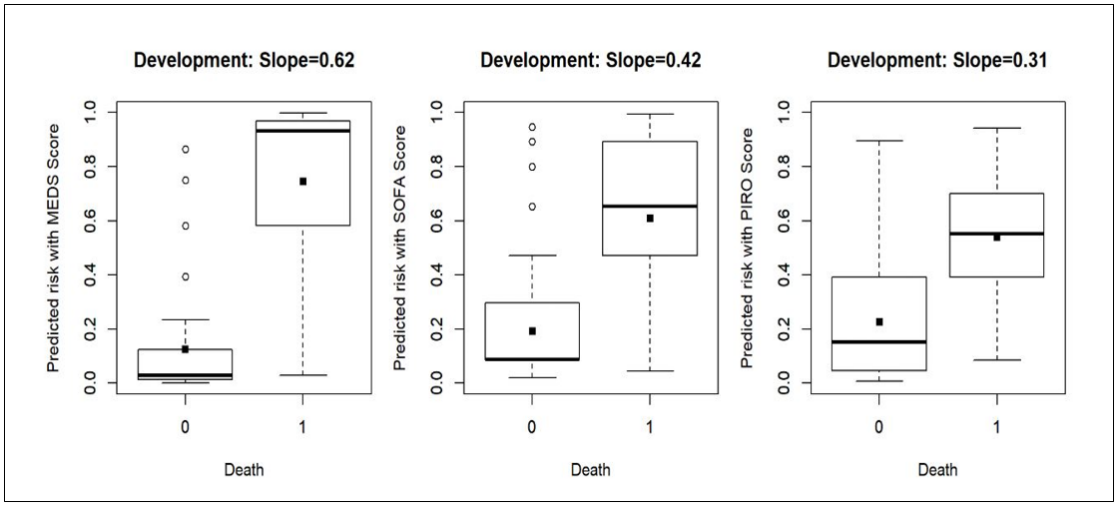
Box plots of predicted probabilities of death in MEDS, SOFA and PIRO scores. The discrimination slope is calculated as the difference between the mean predicted probability of alive and died subjects (solid dots indicate means)

**Figure 2:**
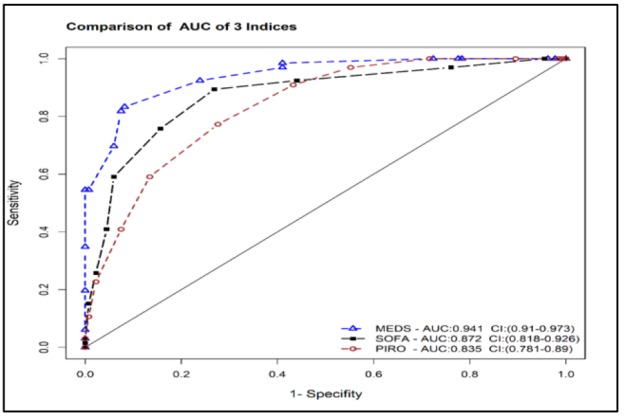
The area under the receiver operator characteristic (ROC) curves of PIRO, MEDS, and SOFA scores in predicting one-month mortality of severe sepsis and septic shock patients. PIRO = Predisposition Insult Response and Organ; MEDS = Mortality in Emergency Department Sepsis; SOFA = Sequential Organ Failure Assessment

***Statistical Analysis***

For quantitative variables with non-normal distribution (evaluated using the Kolmogorov-Smirnov test) median (Interquartile Range=IQR) and for qualitative variables, count (percentage) were used to describe them. The distribution of age, weight, BMI, PIRO, MEDS and SOFA scores among alive and died subjects were compared using T-test and Mann-Whitney test. In addition, the frequency of qualitative variables in the two groups was compared using Chi-square and Fisher's exact test.

We used univariate logistic regression to evaluate the association between MEDS, SOFA and PIRO scores and 30-day mortality among the studied patients. The performance of MEDS, SOFA, and PIRO models for predicting 30-day mortality were evaluated using discrimination and calibration indices. We calculated Brier, Brier_Scaled_ and Nagelkerke’s R^2^ indices for overall performance, C-statistic, discrimination slope, validity indices (sensitivity, specificity, positive predictive value (PPV), negative predictive value (NPV), positive likelihood ratio (PLR) and negative likelihood ratio (NLR)), area under the curve (AUC) and also Box plots calculated for discrimination and calibration-in-the-large, calibration slope and Hosmer-Lemeshow tests were measured for evaluation of calibration. Also, we calculated optimism corrected with the bootstrap method (500) for all performance indices. Data were analyzed using the R software (version 3.4.1). In this study, p<0.05 was considered statistically significant for all statistical tests; yet, we presented the exact p values for all tests. 

## Results

***Baseline characteristics of studied patients***

200 patients with the mean age of 71.03 ± 15.59 (21 - 95) years were studied (61% male). During the 30-day follow up period, 66 (33.0%) patients died (mortality rate = 33.0%; all cases were admitted to ICU). Table 1 compares the baseline characteristics of studied patients between survived and non-survived groups. While the mean age was significantly higher in subjects who died, mean weight and BMI did not show any significant difference between the two groups. Mean PIRO, MEDS and SOFA scores were significantly higher in non-survived cases. Although variables such as the history of underlying diseases were different in the two groups, most of the variables related to admission and vital signs in dead and alive groups were not significantly different. 

There was a significant association between PIRO, MEDS, and SOFA scores with mortality (P<0.001). The Odds ratio of PIRO, MEDS, and SOFA scores in predicting the risk of one-month mortality were 1.9 (95% CI: 1.57 - 2.3), 2.14 (95% CI: 1.73 - 2.65), and 2.1 (95% CI: 1.71 - 2.59), respectively.

**Score performance measurements**

Table 2 summarizes the overall performance, discrimination, and calibration of the scores in predicting the one-month mortality. 

***Overall performance***

Based on Brier, Brier_Scaled _and Nagelkerke’s R^2^ of the models, the best overall performance in predicting one-month mortality belonged to MEDS score. 

***Discrimination***

C-statistic showed that the MEDS score had the highest value in the differentiation between the survived and dead people. Based on Box Plots for predicted probabilities of death in MEDS, SOFA and PIRO scores, the highest discrimination slope belonged to MEDS score (0.62) (Figure 1).

***Calibration***

Area under the ROC curve of PIRO, MEDS, and SOFA scores were 0.83 (95% CI=0.78-0.89), 0.94 (95% CI=0.91-0.97) and 0.87 (95% CI=0.81-0.92), respectively (Figure 2). The optimal cut-off points were 11.5, 5.5 and 6.5 for MEDS, SOFA and PIRO scores, respectively. 

At the cut point of 11.5, MEDS score had a sensitivity of 83.3% (95% CI: 72.1-91.4), specificity of 91.8% (95% CI: 85.8-95.8), PPV of 83.3% (95% CI: 73.0 - 91.4), NPV of 91.8 (95% CI: 85.3-95.8), PLR of 10.15 (95% CI: 5.7-18.1) and NLR of 0.18 (95% CI: 0.1-0.31). 

At the cut point of 5.5, SOFA score had a sensitivity of 75.8% (95% CI: 63.6-85.5), specificity of 84.3% (95% CI: 77.0-90.0), PPV of 70.4% (95% CI: 59.7-81.7), NPV of 87.6 (95% CI: 79.8-92.2), PLR of 4.83 (95% CI: 3.18-7.32), and NLR of 0.28 (95% CI: 0.18-0.44). 

At the cut point of 6.5, PIRO score had a sensitivity of 77.3% (95% CI: 65.3-86.7), specificity of 72.4% (95% CI: 64.0-79.7), PPV of 57.9% (95% CI: 48.3-72.5), the NPV of 86.6 (95% CI: 78.2-90.6), PLR of 2.8 (95% CI: 2.1-3.8), and NLR of 0.31 (95% CI: 0.19-0.49). 

The agreement between predicted mortality using PIRO, MEDS, and SOFA scores and the actual mortality of the study population was determined using Hosmer-Lemeshow (H-L) test. H-L was non-significant for all three scores (Table 2).

## Discussion

The results of the present study showed that MEDS scoring system has better discrimination and performance compared to the other two scoring systems in predicting 30-day mortality of septic shock and severe sepsis patients (area under the curve 0.94). On the best cut-off point, (score=11.5), specificity and sensitivity of this score were 91.8% and 83.3%, respectively.

Results of the present article are in accordance with the AUC of 0.78-0.81 found in previous studies among patients with sepsis (8, 12, 14, 19). In addition, our results are in contrast with a recent study of emergency department sepsis patients (20), which found that MEDS score had an AUC of 0.61 and another study (11) that found an AUC of 0.63 for MEDS in a registry database of patients in the emergency department. 

Differences in the results of various studies could be due to differences in the methods of study. A systematic review of scoring systems in the emergency department showed that there are considerable variation between studies in the mortality rates and inconsistency in the definition of sepsis, severe sepsis, and septic shock. These variations can make valid comparisons problematic (21).

The concept of MEDS score is similar to PIRO and SOFA scores, except that it is specifically designed for emergency patients. In MEDS score calculation, organ dysfunctions receives greater score. In addition to these organ dysfunction parameters, constant data such as age, rapidly terminal comorbid illness, presence of a lower respiratory infection and nursing home residence, is considered in MEDS score (8). Therefore, MEDS score has higher clinical importance (22).

However, this scoring system has some limitations, for example, some data required for MEDS score calculation such as the presence of lower respiratory infection and the number of platelets is not available at the time of triage management. An additional limitation is that in MEDS score calculation, subjective assessment of short-term mortality by the in-charge clinician has a large weighting (14).

The present study showed that a cut off of more than 11.5 points for MEDS score effectively stratified septic shock and severe sepsis patients into two groups, which were significantly different in mortality rate. This cut off point was close to the cutoff point that Chen et al. had calculated in their study (23).

In present study, PIRO and SOFA scores also showed high values of discrimination in predicting 30-day mortality in septic shock and severe sepsis patients (area under the curve 0.835 and 0.872 respectively). However, the discrimination value of these two scorings system was less than MEDS.

Other studies have evaluated the prognostic value of PIRO and SOFA scoring systems in patients with sepsis (13, 23, 24). In Chen's study, PIRO model had an AUC of 0.82 for 28-day mortality (23). In the de Groot's study, among low-risk sepsis patients, PIRO scoring system had an AUC of 0.83; but in higher risk patients, it had an AUC of 0.68 (24).

One of the reasons for the lower prognostic value of PIRO and SOFA scoring systems is that, PIRO model does not require knowledge of the infecting organism and has been adapted specifically for use in the emergency department (25). 

In this study, we used univariate models to evaluate prognostic values of these three indices. It is recommended to evaluate their performance in multivariate models and also externally validate these models in larger studies.

## Limitations

Our study has some limitations. Firstly, this was a mono-center study. Secondly the endpoint was defined as death in 30 days, but the death might have occurred for reasons other than sepsis; and finally, we just compared the performance of these three scores in univariate models. It is recommended to compare these three scores in multivariate prediction models controlling for other patients’ variables to obtain better prediction models for predicting short term mortality.

## Conclusion:

This work shows that MEDS score had an acceptable accuracy in predicting 30-day mortality of patients with severe sepsis and septic shock. 
